# *Salmonella* Infantis Adhesion to Various Surfaces and In Vitro Antimicrobial Efficacy of Commercial Disinfectants

**DOI:** 10.3390/pathogens13110999

**Published:** 2024-11-14

**Authors:** Katja Kranjc, Jana Avberšek, Neva Šemrov, Olga Zorman-Rojs, Darja Barlič-Maganja

**Affiliations:** 1 Faculty of Health Sciences, University of Primorska, Polje 42, 6310 Izola, Slovenia; katja.kranjc@fvz.upr.si; 2 Institute of Microbiology and Parasitology, Veterinary Faculty, University of Ljubljana, Gerbičeva 60, 1000 Ljubljana, Slovenia; jana.avbersek@vf.uni-lj.si; 3 VET.AM.JATA d.o.o., Slomškova ulica 30, 1230 Domžale, Slovenia; neva.semrov@vetam.si; 4 Institute of Poultry, Birds, Small Mammals, and Reptiles, Veterinary Faculty, University of Ljubljana, Gerbičeva 60, 1000 Ljubljana, Slovenia; olga.zormanrojs@vf.uni-lj.si

**Keywords:** *Salmonella* Infantis, disinfectants, adhesion

## Abstract

*Salmonella* Infantis poses a significant challenge in poultry production due to its persistence and resistance to disinfectants. This study investigated the survival of the *S.* Infantis strain on different surfaces and evaluated the efficacy of disinfectants in both preventing and treating biofilms. The survival of the tested *S.* Infantis strain was assessed on plastic and stainless steel surfaces after 24 and 48 h. The minimum inhibitory concentrations (MICs) of five disinfectants were determined, and their antiadhesion effectiveness was evaluated using crystal violet. The efficacy of biofilm treatment was evaluated by cell culturability. The results showed that the adhesion of *S.* Infantis was significantly higher on the plastic surface. The disinfectants were effective at reducing biofilm formation only within the first 24 h. Fresh solutions of disinfectants based on quaternary ammonium compounds exhibited the highest antimicrobial efficacy, while chlorocresol was the most effective for both the prevention and treatment of biofilms. The study results suggest that the presence of plastic surfaces may contribute to the dissemination of *Salmonella*. Additionally, the effectiveness of disinfectants varied based on storage conditions and contact time, while biofilms demonstrated reduced susceptibility compared to planktonic cells. However, given the laboratory scale of this study, further validation on a commercial scale is necessary to confirm these findings.

## 1. Introduction

*Salmonella enterica* subsp. *enterica* serovar Infantis (*S.* Infantis), a globally prevalent serotype associated with foodborne diseases, poses significant public health concerns due to its antimicrobial resistance and ability to form biofilms [1,2]. The antimicrobial resistance of *S.* Infantis is mainly associated with the presence of the pESI plasmid, which confers resistance to tetracyclines, streptomycin, sulfonamides, fluoroquinolones, and trimethoprim [3,4]. The persistence of this pathogen can also be attributed to the protective nature of biofilms, which shield the bacteria from various environmental stressors and disinfectants, thereby reducing the effectiveness of standard cleaning protocols [5]. Moreover, the continuous occurrence of *S.* Infantis in the same agricultural settings indicates its remarkable resilience and tendency for environmental dissemination [6]. The critical sites of *S.* Infantis contamination within poultry facilities and farm environments typically include drinking water systems, floors, and ventilation systems [5,7]. *Salmonella* spp. can form biofilms after attaching to various biotic and abiotic surfaces, such as glass, stainless steel, cement, silicone rubber, polystyrene, and plastic, which are commonly found in poultry production and processing environments [5,8,9]. Several factors influence bacterial adhesion and biofilm formation, including surface characteristics, temperature, and pH, as well as the identity of the serovar [9,10,11,12]. It is important to emphasize that environmental temperature is among the most significant factors influencing biofilm formation. On broiler farms, the temperatures typically range between 18 and 22 °C, which is optimal for in vitro biofilm formation by *Salmonella* spp. This temperature range promotes the expression of biofilm-associated genes, facilitating the persistence of *S.* Infantis in the environment [1]. In addition to temperature, the availability of nutrients plays a crucial role in biofilm formation. Notably, significantly higher levels of biofilm have been observed when conventional laboratory growth media are used [9]. However, the variation in results across different studies underscores the complexity of bacterial adhesion and subsequent biofilm formation, processes that are influenced by a range of interdependent factors.

Given the significant public health risks associated with biofilm-forming *Salmonella* species, there has been a substantial increase in research focused on evaluating the effectiveness of various antimicrobial agents and disinfectants. In this context, biofilms formed by different *Salmonella* strains have been shown to exhibit increased resistance to antibiotics [13] and disinfectants [14] compared to their planktonic counterparts. As with antibiotics, bacteria can develop reduced sensitivity to disinfectants through chromosomal gene mutations or the acquisition of genetic material, such as plasmids [15]. Among these, the globally reported plasmid of emerging *S.* Infantis (pESI) is associated with biofilm formation, antimicrobial resistance, and resistance to heavy metals and disinfectants [4,11,16]. Therefore, the effective use of disinfectants is one of the most important prerequisites for controlling bacterial spread and eliminating foodborne pathogens in both farm and food processing environments. However, the effectiveness of disinfectants depends on several factors, including the active ingredient, its concentration, contact time, surface type, and the cleanliness of the surfaces to which it is applied. As previously shown, environmental temperature, disinfection duration, and the target surface should also be considered for successful disinfection in field situations [17]. It has been observed that bacterial isolates of the same genus and species exhibit varying sensitivities to the same disinfectant. Additionally, disinfectants with similar, though not identical, chemical formulations have shown different levels of efficacy against the same bacterial strains [18].

The most commonly used chemical disinfectants on poultry farms include quaternary ammonium compounds (QACs), oxidizing agents, aldehydes, halogens, and phenols [19]. QACs, a diverse group of cationic surfactants, are commonly used for routine cleaning of noncritical surfaces. While they are effective against Gram-positive bacteria, their efficacy against Gram-negative bacteria, spores, and non-enveloped viruses is limited [20]. In contrast, glutaraldehyde offers a broader spectrum of activity, including sporicidal properties at a suitable pH value. However, once activated, its shelf life depends on the polymerization of glutaraldehyde molecules at alkaline pH and can be as short as 14 days [21]. Chlorine compounds are strong oxidizing agents due to their electronegative nature, but their efficacy can significantly decrease in the presence of organic material [22]. In addition to the other disinfectants, chlorocresol is a chlorinated phenolic compound with broad antimicrobial activity against both bacteria and fungi [23]. Importantly, the chemical stability and disinfection potential of disinfectants are influenced by storage conditions. However, research on the stability of active ingredients, particularly in diluted disinfectant solutions under specific storage conditions, is still limited. In general, manufacturers recommend the daily replacement and preparation of fresh disinfectant dilutions to ensure optimal efficacy [24]. Alongside disinfection procedures, cleaning is the first and most important step in preventing and controlling *S.* Infantis contamination. Electrolyzed water (EW) is an innovative cleaning and disinfecting agent that offers several benefits: it is non-toxic, environmentally sustainable, and effective against several foodborne pathogens. Furthermore, it does not contribute to antimicrobial resistance, making it a valuable tool for enhancing overall sanitation practices [25]. The antimicrobial efficacy of the electrolyzed salt solution can be attributed to one of the three key factors: available chlorine concentration (ACC), pH, and oxidation-reduction potential [26]. However, the ACC tends to decrease with extended storage time and at a higher pH [27], which can lead to reduced antimicrobial activity.

The aim of our study was to test the adhesion potential of a genetically characterized *S.* Infantis strain to surfaces commonly found in food processing facilities, as well as to evaluate the antimicrobial effect of commercial disinfectants. Additionally, the disinfectants were retested after being improperly stored for one year at room temperature and without light protection to determine any potential loss of efficacy.

## 2. Materials and Methods

### 2.1. Adhesion of S. *Infantis* to Different Surfaces

In the present study, the previously characterized *S.* Infantis 323/19 strain [28], originating from broiler chicken, was used. Briefly, this strain belongs to sequence type 32 (ST32) and carries the typical pESI-associated resistance genes: *aadA1*, *sul1*, and *tet*(A). It also harbors genes associated with resistance to QACs (*qacEΔ1*) and mercury (*mer*) but lacks *ars* genes related to arsenic tolerance. This strain was selected due to its demonstrated capacity for high biofilm formation in a previous study [11], making it particularly relevant for our research on biofilms and the efficacy of disinfectants. Prior to the experiments, *S.* Infantis was subcultured aerobically at 37 °C on tryptic soy agar (TSA, Merck KGaA, Darmstadt, Germany). An overnight culture was prepared by inoculating 5 mL of tryptic soy broth (TSB; Oxoid Ltd., Hampshire, UK) with a single bacterial colony and incubating it for 24 h at 37 °C with shaking at 60 rpm. The overnight culture was subsequently diluted 1:100 to obtain a final bacterial concentration of approximately 1 × 10^7^ CFU/mL for use in the experiments.

For the enumeration of *S.* Infantis cells on different surfaces, plastic coupons cut from a feeder (FLUXX Pullet, Big Dutchman, Holland, MI, USA) and stainless steel (AISI 304 and AISI 316) surfaces were used. The assay was performed as previously described, with some modifications [29,30]. Briefly, the coupons of each material were sterilized by autoclaving (121 °C, 15 min) and placed in a 24-well microtiter plate (TPP Techno Plastic Products AG, Trasadingen, Switzerland). Four 10 μL drops of bacterial solution were applied to each coupon and allowed to dry under laminar flow. The coupons were then incubated for 24 and 48 h at 20 °C. As negative controls, 10 μL drops of sterile medium were applied to coupons of each surface and incubated under the same conditions.

After the incubation, a single coupon was transferred to a 50 mL centrifuge tube (Greiner Bio-One GmbH, Kremsmünster, Austria) to which 10 mL of phosphate buffered saline (PBS; Oxoid, Hampshire, UK) was added. The adhered bacterial cells were removed by the sonication method using an ultrasonic bath (room temperature, 10 min; frequency, 37 kHz; and power, 50 W) (Elmasonic P 60 H, Elma Schmidbauer GmbH, Singen, Germany). The number of culturable bacterial cells in suspension was determined using the drop plate method on TSA. The experiment was performed in three technical and two biological replicates. Technical replicates are defined as multiple measurements within the same experiment, in this case using three coupons of the same surface, while biological replicates refer to separate, independently conducted experiments with freshly prepared bacterial culture and new coupons.

### 2.2. Disinfectant Susceptibility Testing

To test the antimicrobial potential, five commonly used disinfectants were applied: Calgonit sterizid P12 DES (Calvatis GmbH, Ladenburg, Germany), DioksiLEK^®^ (Lek Veterina d.o.o, Beltinci, Slovenia), Interkokask^®^ (InterHygiene GmbH, Cuxhaven, Germany), EW (Industrie De Nora S.p.A., Milan, Italy), and Virocid^®^ (CIS LINES N.V., Ieper, Belgium). The concentrated EW solution, containing 4000 ppm of free chlorine at pH 9, was generated using EVA SYSTEM^®^ 100 equipment (Industrie De Nora S.p.A, Milan, Italy), following the manufacturer’s instructions. The active ingredients of the disinfectants are listed in Table 1.

For the disinfectant susceptibility testing, the microdilution method was performed in flat-bottom 96-well clear plates (TPP Techno Plastic Products AG, Trasadingen, Switzerland), with modifications to the previously described method [11]. After filling the wells with 150 μL of TSB, the prepared disinfectant solution (150 μL) was added to the first column. Two-fold serial dilutions were performed across the plate, and each well was then inoculated with 15 μL of the prepared bacterial culture. After 24 h of incubation at 37 °C, the bacterial viability was assessed using PrestoBlue™ Cell Viability Reagent (Life Technologies, Darmstadt, Germany) according to the manufacturer’s instructions. The fluorescence signal was measured using a microplate reader (Infinite F200, Tecan Trading AG, Männedorf, Switzerland). The minimal inhibitory concentrations (MICs) were defined as the lowest concentration of the disinfectant at which no metabolic activity was detected. All the MIC measurements were conducted with three technical and two biological replicates. The control wells contained either culture medium, bacterial suspension, or disinfectant dilutions.

The antimicrobial properties of the disinfectant solutions were also tested after being improperly stored for one year in transparent 50 mL centrifuge tubes (Greiner Bio-One GmbH, Kremsmünster, Austria) at room temperature and without light protection.

### 2.3. Antiadhesion Properties of Disinfectants

As the number of adhered cells was significantly higher on the plastic surface, the antiadhesion properties of the disinfectants were tested on polystyrene. For this assay, subinhibitory concentrations (1/8 MIC) of the disinfectants were tested on the *S.* Infantis 323/19 strain, as previously described [11]. Briefly, 8 wells of a 96-well polystyrene flat bottom microtiter plate (TPP Techno Plastic Products AG, Trasadingen, Switzerland) were inoculated with 200 μL of the prepared suspension (disinfectant dilutions and overnight bacterial culture) and incubated for 24 and 48 h at 20 °C under aerobic conditions. The positive controls contained overnight bacterial culture diluted in sterile TSB, while the negative controls contained disinfectant dilutions in TSB without bacterial culture. After incubation, the suspension was aspirated, and the wells were washed twice with PBS. The plates were then dried at 60 °C for 10 min and stained with 200 μL of 1% crystal violet (CV) solution (Merck KGaA, Darmstadt, Germany) for 15 min. Afterward, the stain was aspirated, and the wells were washed five times under tap water before being dried again at 60 °C for 10 min. The bound CV was released by adding 200 μL of 96% ethanol (Sigma-Aldrich Co., St. Louis, MO, USA). After shaking at 60 rpm for 10 min, the absorbance of the CV solution was measured at 595 nm using a microtiter plate reader (Infinite F200, Tecan Trading AG, Switzerland). The measurements were corrected by subtracting the mean values of the negative controls for each well (ΔA_595_) and interpreted in terms of antiadhesion properties.

### 2.4. S. *Infantis* Biofilm Treatment with Disinfectants

For the biofilm treatment, the disinfectants were prepared in TSB to the final concentrations recommended by the manufacturer for surface disinfection, as well as at their determined MICs (Table 1). TSB was used to mimic the conditions in a nutrient-rich environment, such as the presence of residual organic matter. All the disinfectant solutions were prepared just before the experiment. For the biofilm formation assay, 200 µL of bacterial suspensions was incubated in sealed 2 mL polypropylene microcentrifuge tubes (Eppendorf AG, Hamburg, Germany) at 20 °C for 24 and 48 h. After incubation, the bacterial suspensions were aspirated, and the tubes were washed once with 500 µL of sterile PBS. The cells in the biofilm were then treated with disinfectants at the prepared concentrations for contact times of 15 and 30 min. For the positive control, TSB was used to treat the biofilm. After treatment, the disinfectants were removed, and 200 µL of PBS was added to determine the number of cells in the biofilm as CFU/mL following 10 min sonication in an ultrasonic bath (room temperature, 10 min; frequency, 37 kHz; and power, 50 W) (Elmasonic P 60 H, Elma Schmidbauer GmbH, Singen, Germany). The number of culturable bacterial cells was determined using the drop plate method on TSA. The term culturable refers to cells that were able to form colonies under the experimental conditions. The experiment was conducted in three technical and three biological replicates.

### 2.5. Statistical Analysis

To validate the differences in (a) the number of adhered *S.* Infantis cells on different surfaces, (b) the absorbance of the CV solution (A_595_) for each experimental set of wells when assessing the antiadhesive effect of disinfectants, and (c) the number of cultivable cells in the biofilm after treatment with disinfectants, one-way ANOVA was used for normally distributed variables, and a Kruskal–Wallis test was applied for non-normally distributed variables. In terms of the post hoc tests, the Bonferroni correction was employed. When comparing only two groups of data, such as the number of cells at two different time points, a *t*-test was used for normally distributed variables, while the Mann–Whitney U test was used for non-normally distributed variables. The analysis was performed using SPSS software version 26 (IBM, Armonk, NY, USA), with statistical significance set at *p* < 0.05.

## 3. Results

### 3.1. Survival of S. *Infantis* on Different Surfaces

**Figure 1:**
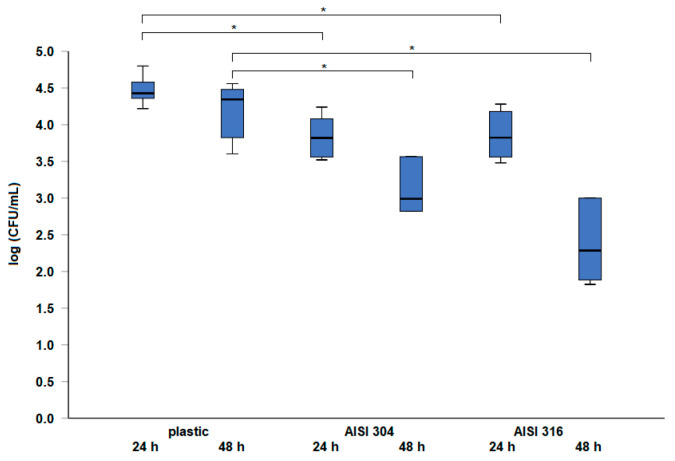
Survival of *S.* Infantis 323/19 on different surfaces (plastic cut from a feeder and stainless steel types AISI 304 and AISI 316) after incubation at 20 °C for 24 and 48 h. * indicates a statistically significant difference (*p* < 0.05).

The number of culturable cells after 24 and 48 h of incubation at 20 °C on different surfaces is presented in Figure 1. The results showed that the number of *S.* Infantis cells was significantly higher on the plastic surface after both 24 and 48 h of incubation (*p* < 0.05). However, no significant difference was observed in the number of cells between the two stainless steel types, AISI 304 and AISI 316.

### 3.2. Antimicrobial Effect of Disinfectants

The antimicrobial effect of the disinfectants, determined as MICs, are presented in Table 1, alongside the concentrations recommended by the manufacturer for surface disinfection. Among the freshly prepared solutions, Virocid^®^ exhibited the highest antimicrobial effect, followed by Calgonit sterizid P12 DES and Interkokask^®^. The MIC for EW was also found to be lower than the concentration of free chlorine recommended by the manufacturer. In contrast, the MIC for DioksiLEK^®^ was the only one that was higher than the recommended concentration for surface disinfection.

However, when improperly stored at room temperature and unprotected from light, the effectiveness of all the disinfectants decreased, except for Interkokask^®^, which retained the same MIC value. In the case of improperly stored EW, the tested concentrations of free chlorine showed no antimicrobial effect on *S.* Infantis.

### 3.3. Antiadhesion Effect of Disinfectants on Polystyrene

**Figure 2:**
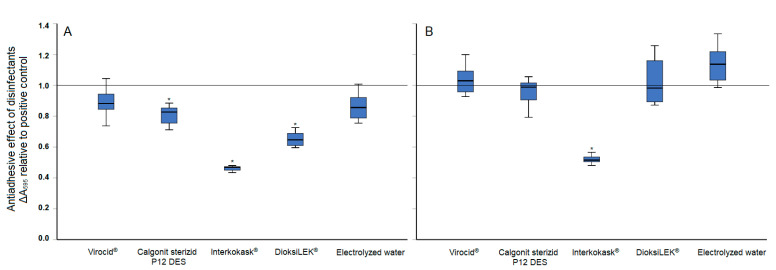
Antiadhesion effect of disinfectants in subinhibitory concentrations on *S.* Infantis 323/19 after incubation at 20 °C for (**A**) 24 h and (**B**) 48 h. * indicates a statistically significant difference (*p* < 0.05) compared to positive control.

The antiadhesion effect of freshly prepared disinfectants at subinhibitory concentrations is shown in Figure 2. The results indicated that even at 1/8 MIC, the majority of the tested disinfectants (three out of five) significantly (*p* < 0.05) reduced biofilm formation within the first 24 h. However, after a longer incubation period (48 h), only Interkokask^®^ exhibited significant effectiveness (Figure 2B). Additionally, following improper storage of the disinfectant solutions, Interkokask^®^ was the only disinfectant that maintained its antiadhesion effect, with ΔA_595_ values lower than those of the positive control for 15.38 ± 4.57% after 24 h and 14.32 ± 4.77% after 48 h.

### 3.4. Biofilm Treatment with Disinfectants

The treatment of 24 h old *S.* Infantis biofilm with the determined MICs of the disinfectants was generally ineffective, except for Interkokask^®^, which significantly (*p* = 0.001) reduced the culturability of cells in the biofilm by 11.99 ± 1.17% (Table 2).

**Figure 3:**
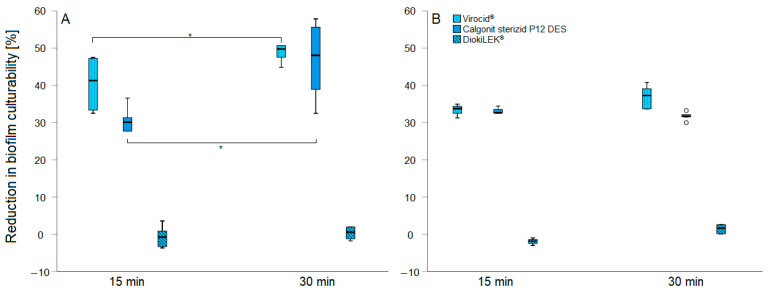
The reduction in culturability of *S.* Infantis 323/19 in (**A**) 24 h and (**B**) 48 h old biofilms, following treatment with disinfectants at recommended concentrations for 15 or 30 min. * indicates a statistically significant difference (*p* < 0.05).

The efficacy of the freshly prepared disinfectants at their recommended concentration is shown in Figure 3. The data are presented as the relative difference in the reduction in culturable bacterial cells after the treatments, compared to the untreated control. The number of culturable cells in the positive control was significantly higher (*p* = 0.009) in the 48 h biofilm compared to the 24 h old biofilm (8.23 ± 0.19 vs. 7.86 ± 0.31 log CFU/mL). When treating the 24 h old biofilm, Interkokask^®^ (2% (*v*/*v*)) and EW (4000 ppm of free chlorine) were completely effective in eliminating cell culturability by 100%, followed by Calgonit sterizid P12 DES, Virocid^®^, and DiokiLEK^®^ (Figure 3). A statistically significant difference (*p* < 0.05) was observed between the contact times for Calgonit sterizid P12 DES and Virocid^®^ when treating the 24 h old biofilm, with greater effectiveness at a 30 min contact time (Figure 3A). However, when treating the 48 h old biofilm, no significant differences were observed between the contact times used (Figure 3B). The counts of the culturable cells after treatment with DioksiLEK^®^ did not differ significantly from those of the positive control. When comparing the effectiveness of the treatments on the 24 h and 48 h old biofilms, Virocid^®^ was the only disinfectant that showed a significantly higher efficacy (*p* = 0.004) against the younger biofilm but only at a 30 min contact time (Figure 3).

Given the complete effectiveness of freshly prepared solutions of Interkokask^®^ and EW, both disinfectants were also tested after improper storage. While Interkokask^®^ remained completely effective, EW did not significantly reduce the number of culturable cells compared to the positive control (Table 2).

## 4. Discussion

The adhesion and survival of bacteria on different surfaces can vary significantly. In this study, we investigated the survival of *S.* Infantis on plastic and stainless steel surfaces following two incubation times at a temperature favorable for biofilm formation. The most common sources of *Salmonella* contamination in poultry production, as identified through a meta-analysis, include the hatchery, litter, faeces, the internal and external poultry house environment, feed, chicks, and drinkers [31]. Heyndrickx et al. (2002) reported *Salmonella* contamination in all examined flocks (n = 18) during hygiene control, with feed trays and the feed within them identified as positive sampling sites [32]. Therefore, regular monitoring of water and feed is crucial, as these are potential sources of *Salmonella* introduction. The significantly higher survival rate of *S.* Infantis on the plastic surface compared to stainless steel (AISI 304 and AISI 316) in our study suggests that plastic may provide a more conducive environment for bacterial persistence. These findings indicate that plastic surfaces can serve as significant reservoirs of contamination, thereby contributing to the spread of *Salmonella* on poultry farms. Previous studies have shown that plastic surfaces, due to their hydrophobic nature and surface roughness, tend to promote greater bacterial adhesion and biofilm formation compared to smoother, hydrophilic surfaces, like stainless steel [33,34]. However, the influence of surface material on bacterial adhesion can vary depending on the experimental conditions used; for instance, the type of surface may not always have a significant effect on pathogen adherence [35]. Since the surfaces were not characterized in the present study, our understanding of how factors such as hydrophobicity or surface roughness may have influenced *S.* Infantis adhesion is limited. Furthermore, our results showed that *S.* Infantis can survive for at least 48 h on all tested surfaces, with levels ranging from 4.04 ± 0.45 log CFU/mL on plastic to 2.38 ± 0.52 log CFU/mL on AISI 316 stainless steel. This highlights the importance of considering the surface material when evaluating contamination risks, particularly in environments where biofilm formation can promote bacterial survival. Although adhesion rates on both AISI 304 and AISI 316 stainless steel surfaces were lower compared to those on plastics, there was no significant difference between the two stainless steel types. However, AISI 316 offers enhanced corrosion resistance due to its molybdenum content, which may contribute to its long-term durability in harsh environments [36]. Since *Salmonella* primarily spreads among broilers via the fecal–oral route, contaminated surfaces can harbor the bacteria for extended periods, contributing to an ongoing cycle of contamination. Additionally, if contaminated surfaces are not adequately cleaned and disinfected, they can facilitate the transmission of *Salmonella* to new flocks introduced into the facility.

Considering the crucial role of cleaning and disinfection in reducing infection in new flocks and preventing bacterial persistence in facilities, this study evaluated the efficacy of five commercially available disinfectants. The antimicrobial potential of disinfectants can vary significantly based on their active ingredients and modes of action, which in turn affects their overall effectiveness in controlling bacterial contamination. In our study, all tested disinfectants demonstrated antimicrobial efficacy, with fresh solutions of Virocid^®^ and Calgonit sterizid P12 DES exhibiting the highest effectiveness at very low concentrations. Consistent with these findings, products containing a mixture of formaldehyde, glutaraldehyde, and QACs performed significantly better in eliminating *Salmonella* contamination under field conditions compared to oxidizing products [37,38]. However, when stored improperly at room temperature and exposed to light, the efficacy of most disinfectants decreased, except for Interkokask^®^, which maintained the same MIC value. This decrease in disinfectant efficacy emphasizes the importance of proper storage conditions to preserve their antimicrobial properties or to ensure a fresh preparation before use. The consistent performance of Interkokask^®^, regardless of storage conditions, suggests that it is the most reliable option for long-term use. In line with our findings, chlorocresol-based disinfectants have been reported to consistently achieve high *Salmonella* kill rates under both wet and dry test conditions [39]. Compared to conventional disinfection methods, EW offers several advantages, including shorter treatment times, ease of application, lower costs, and greater effectiveness in removing biofilms even after a short contact time [40]. Moreover, a previous study has shown that EW effectively reduced *Campylobacter jejuni* populations in chickens and prevented cross-contamination in the processing environments [41]. Although basic EW has limited sterilization properties, clinical practice suggests that its consumption can greatly improve gastrointestinal symptoms [42]. However, our study showed that the antimicrobial and antibiofilm efficacy of EW was completely lost over time, highlighting the necessity for its fresh preparation to effectively control *S.* Infantis contamination. Furthermore, the use of disinfectants with different modes of action throughout poultry processing is recommended, as this approach can reduce bacteria’s ability to adapt and develop resistance to antimicrobials [18].

It is acknowledged that data on MICs for disinfectants are not always reliable indicators for their effectiveness in the presence of organic matter and/or biofilm [43]. A study evaluating commonly used farm disinfectants in both wet and dry models of *Salmonella* contamination found that some disinfectants, which had demonstrated effectiveness in standardized tests, were unable to completely eradicate the pathogen [39]. This demonstrates that bacterial cells can be protected from effective biocide concentrations due to factors, such as organic matter, surfaces, biofilms, or improper disinfectant application. Additionally, it is important to note that such selective pressure can significantly impact the survival of bacterial populations, reducing their susceptibility to antimicrobial agents [44]. Biofilms play a crucial role in bacterial survival, persistence, and resistance to environmental stressors, including cleaning and disinfection processes. Given *S.* Infantis’s ability to form biofilms on various surfaces, we tested the efficacy of different disinfectants in both preventing adhesion and removing adhered cells. All the experiments were conducted in TSB, the most commonly used laboratory culture medium for biofilm formation, which has been reported to yield higher biofilm levels compared to certain food matrices [9]. Moreover, TSB at varying concentrations has been previously used to simulate the presence of organic material [45], a condition typically mimicked using bovine serum albumin (BSA) solution [46]. The presence of organic matter can hinder the disinfectants from coming into direct contact with the target microorganisms or interact chemically with the active compounds, thus neutralizing or reducing their effectiveness. Although subinhibitory concentrations of disinfectants were used in the antiadhesion assay, the majority of them showed an effect on reducing biofilm formation within the first 24 h. However, only incubation with Interkokask^®^ resulted in a significant reduction (*p* < 0.05) in biofilm levels even after 48 h, compared to the positive control.

Multi-drug-resistant (MDR) *Salmonella* species, including *S*. Infantis, exhibit high resistance to standard cleaning and disinfection protocols, with an increased ability to persist in the broiler farm environment [47]. A study assessing the susceptibility of diverse *Salmonella* strains to antibiotics and benzalkonium chloride revealed that genes conferring resistance to QACs through the efflux pump system (*qacEΔ1*), associated with the pESI-like plasmid [28], confer disinfectant resistance. Importantly, QAC disinfectant products can induce the co-expression of antibiotic resistance genes [48]. The general ineffectiveness of disinfectants at MIC concentrations against 24 h old biofilm in this study highlights the challenge of eradicating established biofilms at such low concentrations. These findings suggest that cells in biofilms are less susceptible to disinfectants compared to planktonic cells, as previously demonstrated [49,50]. Fresh solutions of Interkokask^®^ and EW were found to be fully effective against both 24 and 48 h old biofilms, regardless of contact time. When comparing the efficacy based on the contact time, Calgonit sterizid P12 DES and Virocid^®^ showed significantly higher performance (*p* < 0.05) with longer exposure times but only in the treatment of 24 h old biofilms. In contrast, contact time did not affect the survival rate of *S.* Infantis when treating 48 h old biofilms. This may be explained by the higher number of culturable cells present in the 48 h biofilm, as well as the increased levels of protective polysaccharides that accumulate over time [51]. Conversely, no statistically significant differences in disinfectant effectiveness were observed when comparing treatments of 24 and 48 h old biofilms, except for Virocid^®^, which was more effective in reducing the survival rate of cells in the 24 h old biofilm. In the case of DioksiLEK^®^, which showed the lowest reduction rates, this could be due to the fact that the concentrations of other disinfectants used in this study were several times higher than their respective MIC values. Notably, the incomplete effectiveness in eliminating the biofilms suggests that the presence of viable cells may exert selective pressure, potentially leading to the development of populations resistant to multiple antimicrobial agents. Furthermore, the survived biofilm cells serve as a reservoir of contamination, facilitating the potential re-colonization of surfaces, equipment, and the surrounding environment.

Nevertheless, several limitations of this study should be acknowledged. The primary limitation regarding the survival of *S.* Infantis on different surface materials is the lack of detailed surface characterization. This may affect the accuracy and applicability of the findings related to bacterial adhesion and survival on different surfaces. Additionally, only one strain of *S.* Infantis was used, which limits the generalizability of the results; working with multiple strains would provide more representative data. Another limitation is the absence of a neutralizing agent, which could have effectively stopped the action of the disinfectant post-application. Neutralizers are critical for accurately assessing the antimicrobial efficacy of disinfectants, as they prevent any residual activity that could artificially influence results during the microbial recovery stages. Furthermore, follow-up assessments were not conducted to evaluate the impact of extended exposure to the disinfectants. Through these studies, we could assess the persistence of disinfectant efficacy, as well as microbial adaptation or regrowth after the initial application. Therefore, future research will focus on a more complex model that better reflects actual environmental scenarios, where inaccessibility to certain areas for cleaning or/and disinfection remains a significant challenge.

## 5. Conclusions

*S.* Infantis poses a significant challenge in poultry production due to its persistence in the environment, ability to form biofilms, and resistance to antimicrobials and disinfectants. The results of this study provide valuable insights into the survival of *S.* Infantis on different surfaces and the efficacy of various commercially used disinfectants. Our findings demonstrate that plastic surfaces promote higher bacterial adhesion and survival rates compared to stainless steel. Additionally, the antiadhesion potential of most disinfectants was significantly diminished in older biofilms, suggesting that established biofilm communities are more resilient and less susceptible to disinfectant action. Overall, the chlorocresol-based disinfectant demonstrated strong antiadhesion properties and was fully effective in treating biofilms. However, to obtain more reliable insights, the efficacy of disinfectants should be assessed under actual field conditions. Further research could also explore the mechanisms behind the varying efficacy of disinfectants and the potential for developing more effective biofilm control strategies.

## Figures and Tables

**Table 1:** Minimal inhibitory concentrations (MICs) of disinfectants against *S.* Infantis 323/19.

Disinfectant	Active Ingredients	MIC		Recommended Dose ^1^
		Fresh solution	Old solution	
Calgonit sterizid P12 DES	glutaraldehyde and QACs	0.00156% (*v*/*v*)	0.03125% (*v*/*v*)	0.5% (*v*/*v*)
DioksiLEK^®^	chlorine dioxide solution	3.5% (*v*/*v*)	14% (*v*/*v*)	0.2–1% (*v*/*v*)
Interkokask^®^	chlorocresol	0.0625% (*v*/*v*)	0.0625% (*v*/*v*)	2% (*v*/*v*)
Electrolyzed water	free chlorine	750 ppm	>2000 ppm	4000 ppm
Virocid^®^	quaternary ammonium, glutaraldehyde and isopropanol	0.000195% (*v*/*v*)	0.0078% (*v*/*v*)	0.25–0.5% (*v*/*v*)

^1^ Recommended concentration by the manufacturer for the surface disinfection. Legend: QACs—quaternary ammonium compounds.

**Table 2:** Treatment of 24 h old *S.* Infantis 323/19 biofilm with minimal inhibitory concentrations (MICs) and improperly stored disinfectant solutions.

Disinfectant	Treatment with MICs	Treatment with Improperly Stored Solutions	
		15 min	30 min
Calgonit sterizid P12 DES	0.63 ± 1.01	(-)	(-)
DioksiLEK^®^	−2.35 ± 2.38	(-)	(-)
Interkokask^®^	11.99 ± 1.17	100	100
Electrolyzed water	2.45 ± 3.93	0.05 ± 2.43	0.67 ± 1.17
Virocid^®^	−1.36 ± 2.77	(-)	(-)

The data are presented as the percentage [%] reduction in the culturability of cells in biofilm compared to the positive control. (-) indicates that the disinfectant was not tested.

## Data Availability

The data presented in this study are available on request from the corresponding author.
